# In utero protein restriction causes growth delay and alters sperm parameters in adult male rats

**DOI:** 10.1186/1477-7827-9-94

**Published:** 2011-06-24

**Authors:** Fabíola C Toledo, Juliana E Perobelli, Flávia PC Pedrosa, Janete A Anselmo-Franci, Wilma DG Kempinas

**Affiliations:** 1 Graduate Program in Cell and Structural Biology, Institute of Biology, University of Campinas - UNICAMP, Campinas, SP, Brazil; 2 Department of Morphology, Institute of Biosciences, UNESP - Universidade Estadual Paulista, Botucatu, SP, Brazil; 3 Department of Morphology, Stomatology and Physiology, School of Dentistry, University of São Paulo - USP, Ribeirão Preto, SP, Brazil

## Abstract

**Background:**

Recent studies have supported the concept of "fetal programming" which suggests that during the intrauterine development the fetus may be programmed to develop diseases in adulthood. The possible effects of *in utero *protein restriction on sexual development of rat male offspring were evaluated in the present study.

**Methods:**

*Pregnant *Wistar rats were divided into two experimental groups: one group treated with standard chow (SC, n = 8, 17% protein) and the other group treated with hypoproteic chow (HC, n = 10, 6% protein) throughout gestation. After gestation the two experimental groups received standard chow. To evaluate the possible late reproductive effects of *in utero *protein restriction, the male offspring of both groups were assessed at different phases of sexual development: prepubertal (30 days old); peripubertal (60 days old); adult (90 days old). Student's t-test and Mann-Whitney test were utilized. Differences were considered significant when p < 0.05.

**Results:**

We found that *in utero *protein restriction reduced the body weight of male pups on the first postnatal day and during the different sexual development phases (prepubertal, peripubertal and adult). During adulthood, Sertoli cell number, sperm motility and sperm counts in the testis and epididymal cauda were also reduced in HC. Furthermore, the numbers of sperm presenting morphological abnormalities and cytoplasmic drop retention were higher in HC.

**Conclusions:**

In conclusion, *in utero *protein restriction, under these experimental conditions, causes growth delay and alters male reproductive-system programming in rats, suggesting impairment of sperm quality in adulthood.

## Background

In recent years, an increasing amount of evidence has supported the idea that disturbances occurring in critical periods of fetal development may determine permanent or long-term changes in the physiology or morphology of an organ [1]. There are several studies relating the gestational environment to the late effects on the body composition of animals, evidencing this phase as a critical period for the genesis of diseases. These studies support the concept of "fetal programming", which suggests that, during intrauterine development, the fetus may be programmed to develop diseases during adulthood [2,3]. According to this paradigm, the susceptibility to diseases (including reproductive diseases and dysfunctions) is influenced by diet, environmental exposure to toxic agents and stress during the fetal and neonatal periods [4].

Development and growth in the intra- and post-uterine periods depend on the nutritional, hormonal and metabolic environment provided by the mother during gestation and lactation [5]. The physiological processes that occur during the gestational period, including augmented blood volume, increased tissue, and growth of the placenta and fetus require a higher contribution of nutrients (proteins, vitamins, mineral salts etc.). Protein contribution is known to be essential for the maintenance and success of a pregnancy [6].

One of the most important types of fetal programming is the profile of nutritional status in early life, with low birth weight being a marker of poor fetal nutrition [7]. In fact, prenatal malnutrition is the most frequently studied fetal programming model [2,3]. Based on the literature, maternal protein restriction, alone or combined with energy restriction during gestation, leads to consistent diminution of fetal growth in many species. According to Desai et al. [8], maternal protein deprivation not only affects the body growth of pups, but also changes their body composition, selectively affecting their organs. Those authors also stated that the postnatal weight recovery of organs does not necessarily signify a morpho-functional recovery.

Most studies in this area are carried out to investigate the cardiovascular system [9-12]. Studies correlating maternal malnutrition effects with the development of the male reproductive system are scarce. The importance of prenatal and neonatal factors, especially the nutritional status, in the programming of the reproductive function has only become accepted in recent decades [13-17]. Currently, some authors have focused on verifying the late effects of nutrient restriction during pregnancy and lactation on sexual development and reproductive function of offspring in adulthood [16,18,19].

Birth weight is a crucial indicator of the degrees of compromise and development of the individual [17]. Studies have shown that *in utero *protein restriction causes low birth weight and compromises the establishment of puberty in male and female rats [16,17,20]. Puberty in the male rat begins around the age of 50 days [21]. At between 75 days (maximum sperm production) and 100 days of age (maximum concentration of sperm stored in the epididymis cauda), the animals are supposed to reach plain sexual maturity [21,22]. According to Clegg [23] and Ojeda et al. [24], the male postnatal sexual development is divided into 4 phases: (1) neonatal, from postnatal day (PND) 1 to 7; (2) infantile, from PND 8 to 21; (3) juvenile, PND 22 to 35; and (4) peripubertal, from PND 36 to 55 or 60.

Some authors have reported reduced testicular weight and increased expression of androgen receptors in the testicle of rat offspring that had been subjected to food or protein restriction during gestation [25,26]. Genovese et al. [27] reported a smaller number of Sertoli cells in adult animals that underwent malnutrition during fetal and prepubertal life. In addiction, nutritional status is also known to cause a variety of effects on the endocrine system [28-31]. Changes in levels of corticosterone, testosterone, estradiol and LH were reported in both mothers and progeny after food restriction [16,26]. However, the influence of protein restriction on sperm production, storage and quality remains poorly understood.

Based on the relationship between malnutrition and programming of the male reproductive system, the present study aimed to evaluate the possible late effects of *in utero *protein restriction on the sexual development and reproductive function of adult male rats, focusing on sperm production, storage and quality.

## Methods

The experimental protocol followed the Ethical Principles in Animal Research of the Brazilian College of Animal Experimentation and was approved by the Biosciences Institute/UNESP Ethics Committee for Animal Experimentation (protocol n° 10/08-CEEA).

### Animals

Adult female (60 days of age, *n *= 20) and male (90 days of age, *n *= 10) Wistar rats were supplied by the Central Biotherium of São Paulo State University - UNESP and were housed in polypropylene cages (43 cm × 30 cm × 15 cm) with laboratory-grade pine shavings as bedding. Rats were maintained under controlled temperature (23 ± 1°C) and lighting conditions (12:12-h photoperiod, light period beginning at 0700 h). Two non-gravid female rats were mated with one male, during the dark portion of the lighting cycle, and the day of sperm detection in the vaginal smear was considered day 0 of gestation (gestation day 0 - GD 0). The gravid females were randomly assigned between the experimental groups and housed individually in cages. Pregnant female rats were divided into two experimental groups: one treated with standard chow (SC, n = 8, 17% protein) and the other treated with hypoproteic chow (HC, n = 10, 6% protein) throughout gestation (GD0-GD21). During the lactation period the two groups received standard chow. After lactation, pups also received water and standard chow *ad libitum*. The low protein and standard diets, whose compositions are described in Table 1, were prepared in the Experimental Laboratory of the School of Medicine - UNESP, São Paulo State University, Botucatu, SP, Brazil. The diets were isocaloric and normosodic (0.20%).

**Table 1:** Diet Compositions (g/kg)

Ingredient	Hypoproteic chow (6% protein)	Standard chow (17% protein)*
Cornstarch	480	397
Casein (84%)	71.5	202
Dextrinized cornstarch (90-94%)	159	130.5
Sucrose	121	100
Soybean oil	70	70
Fiber	50	50
Mineral mixture **	35	35
Vitamin mixture **	10	10
L-cystine	1	3
Choline bitartrate	2.5	2.5

* Diet formulated for the growth, pregnancy and lactational phases of rodents -AIN- 93G [61]

** According to AIN-93G

### Evaluation of the reproductive development of male offspring

On post-natal day 1 (PND 1) male pups were weighed and the litters were reduced to 10 pups, in order to maintain a similar number of males and females. To evaluate the possible late reproductive effects of *in utero *protein restriction, the male offspring of both experimental groups (SC and HC) were assessed at different phases of sexual development: prepubertal (phase I, n = 10 per group) pups sacrificed at the age of 30 days; peripubertal (phase II, n = 10 per group) pups sacrificed at 60 days old; adult (phase III, n = 20 per group) pups sacrificed at 90 days of age. In phase III a larger number of rats was used because more parameters were assessed in these animals.

### Collection and weighing of organs

At the specified ages, 10 pups per experimental group were anaesthetized with ether and killed by decapitation. The left testis and epididymis, seminal vesicle (without the coagulating gland and full of secretion), left deferens duct and ventral prostate were removed and their weights (absolute and relative to entire body) were determined. The right testis and epididymis from experimental animals were collected for processing and histological and / or morphometric analysis.

Phase III blood samples were collected from cervical vessels, ruptured by decapitation, to determine sexual hormone levels. Other pups in phase III (n = 10 per experimental group) were killed to collect the left testis to measure intratesticular testosterone and sperm from the deferens duct to analyze the sperm morphology and motility. The right testis and epididymis were collected for determination of the germ cell number.

### Histological evaluation

The right testis and epididymis were removed and fixed in Alfac fixing solution (80% ethanol, formaldehyde and glacial acetic acid, 8.5: 1.0: 0.5, v ⁄ v) for 24 h. The pieces were embedded in paraffin wax and sectioned at 5 μm. The sections were stained with hematoxylin and eosin, and observed by light microscopy, in a blind assay for histological examination.

One hundred seminiferous tubule cross sections per animal were evaluated as to normal or abnormal histological aspect. The whole epididymal histological sections were also evaluated.

### Morphometric analysis

#### Sertoli cell number

To assess the possible effects of *in utero *protein restriction on the proliferation of Sertoli cells, the nuclei of Sertoli cells were counted in 20 seminiferous-tubule cross-sections per rat in phase III (n = 8 per group) at stage VII of spermatogenesis, as classified by Leblond & Clermont [32].

#### Stereological analysis

In addition to the histological evaluation, a more detailed assessment of the proximal cauda epididymis was carried out by stereological analysis, according to the method described by Weibel [33]. Longitudinal sections of epididymal cauda were obtained from experimental animals at 60 and 90 days old and assessed with the aid of a Leica ^® ^light microscope at 200× magnification, 10 fields per animal. A test system consisting of a graticule with 120 points and 60 lines was employed to obtain the relative proportions of luminal, epithelial and interstitial compartments.

#### Degree of maturation of the germinal epithelium

In order to evaluate the maturation degree of the seminiferous epithelium, 100 cross-sections of seminiferous tubules per animal (n = 8 per group) in phases I, II and III, were evaluated randomly, using the adapted method of assigning values according to the type of mature germ cell most numerous in the tubular epithelium [34]: degree 1: spermatocytes I or II; degree 2: young spermatids with rounded nucleus (stage 1 to 8 of spermiogenesis); degree 3: spermatids in maturation phase, with ovoid or elongated nucleus (stage 9 to 14 of spermiogenesis); degree 4: spermatids in maturation phase, with elongated nucleus (stage 15 to 18 of spermiogenesis); degree 5: mature spermatids (stage 19 of spermiogenesis) in small quantity; degree 6: mature spermatids (stage 19 of spermiogenesis) in average amount; degree 7: mature spermatids (stage 19 of spermiogenesis) in larger amount. The number of seminiferous tubules in each degree was multiplied by its degree, and then the values were added and divided by 100, resulting in the "average degree".

### Sperm counts, daily sperm production and sperm transit time through the epididymis

Homogenization-resistant testicular spermatids (stage 19 of spermiogenesis) and sperm in the caput ⁄ corpus epididymis and cauda epididymis were counted as described previously by Robb et al. [21], with adaptations specified by Fernandes et al. [35]: the right testis, decapsulated and weighed soon after collection, was homogenized in 5 mL of NaCl 0.9% containing Triton X100 0.5%, followed by sonication for 30 sec. After a 10-fold dilution a sample was transferred to a Neubauer chamber (four fields per animal), preceding a count of mature spermatids. To calculate daily sperm production (DSP) the number of spermatids at stage 19 was divided by 6.1, which is the number of days these spermatids are present in the seminiferous epithelium. Caput/corpus and cauda epididymis portions were counted as described for the testis. The sperm transit time through the epididymis was determined by dividing the number of sperm in each portion (caput⁄corpus and cauda epididymis) by the DSP.

### Analysis of sperm motility

Sperm were obtained from the right deferens duct of rats in phase III (n = 10 per group) and immediately diluted in 2 ml of modified HTF medium (Human Tubular Fluid, IrvineScientific^*®*^), pre-heated to 34°C. An aliquot of 10 μL was placed in a Makler chamber (Irvine, Israel) and analyzed under a light microscope, at 200X magnification. One hundred sperm were evaluated per animal and classified for motility into: type A: mobile, with progressive trajectory; type B: mobile, with non-progressive trajectory; type C: immobile.

### Analysis of sperm morphology

For the evaluation of sperm morphology, the left deferens duct was sectioned at its extremities and washed with the aid of a syringe, coupled to a needle, containing 1.0 mL of formol-saline solution. The washed product was collected in an Eppendorf tube and, soon after, smears were prepared on histological slides and left to dry in open air.

Two hundred spermatozoa (heads only or intact sperm) per animal were evaluated for head and/or flagellar defects by phase-contrast microscopy (200X total magnification) [36]. These were also classified as the presence or absence of a cytoplasmic droplet.

### Hormonal assay

After decapitation, blood was collected from the ruptured cervical vessels in a tube for the determination of serum testosterone, luteinizing hormone (LH) and follicle-stimulating hormone (FSH) levels. The serum was obtained after blood centrifugation (2400 rpm, 20 min, 4°C) and stored at -20°C until the moment of hormonal determination. Serum testosterone levels were determined by double-antibody radioimmunoassay using a Coat-A-Count_ kit (Diagnostics Products Corporation, Los Angeles, CA, USA). Serum LH and FSH levels were determined using specific kits provide by the National Institute of Arthritis, Diabetes, Digestive and Kidney Diseases (NIADDK; Baltimore, USA).

The left testis of each animal was removed and decapsulated, and the parenchyma was sliced into 50-mg pieces. Each piece was weighed and placed into a 1.5-mL microfuge tube containing 1.0 mL of Medium 199 (M199). The M199 was buffered with 0.71 g/L sodium bicarbonate (NaHCO3) and 2.1 g/L Hepes, and contained 0.1% BSA (Schwartz-Mann, Orangeburg, NY) and 25 mg/L soybean trypsin inhibitor, pH 7.4. After centrifugation (5 min, 10000 rpm), the medium was frozen at -70 ◦C until the testosterone assay was performed using a Coat-A-Count_ kit (Diagnostics Products Corporation, Los Angeles, CA, USA).

### Statistics

Values were expressed as mean ± SEM and medians. To compare results between the experimental groups, the Student's t-test and Mann-Whitney test were used. Differences were considered significant when p < 0.05.

## Results

At PND1 and at different studied phases of development (30, 60 and 90 days old) the HC group animals presented a statistically significant reduction in body weight (Table 2). The absolute weights (mg) of the rat testis at 30 days old (HC Group: 163.67 ± 13.97*; SC Group: 200.85 ± 8.29) and the deferens duct (mg) of the 90-day-old rats (HC Group: 84.97 ± 2.75*; SC Group: 95.85 ± 3.29) were statistically lower in the HC than the SC group (values expressed as mean ± SEM, *p < 0.05). The relative and absolute weights of the other sexual organs were similar between the experimental groups at PND 30, 60 and 90 (data not shown).

**Table 2:** Body weight (g) of male pups on PND1 (n = 10 litters per experimental group), on PND30, 60 and 90 (HC group: n = 10 pups per age; SC group: n = 8 pups per age)

Body weight (g)	HC Group	SC Group
DPN 1	6.07 ± 0.12*	6.87 ± 0.18
DPN 30	57.53 ± 2.65*	70.88 ± 2.01
DPN 60	257.42 ± 8.81*	287.40 ± 5.67
DPN 90	351.53 ± 11.93*	407.15 ± 9.75

Values expressed as mean ± SEM. Student's t test, *p < 0.05

**Figure 1:**
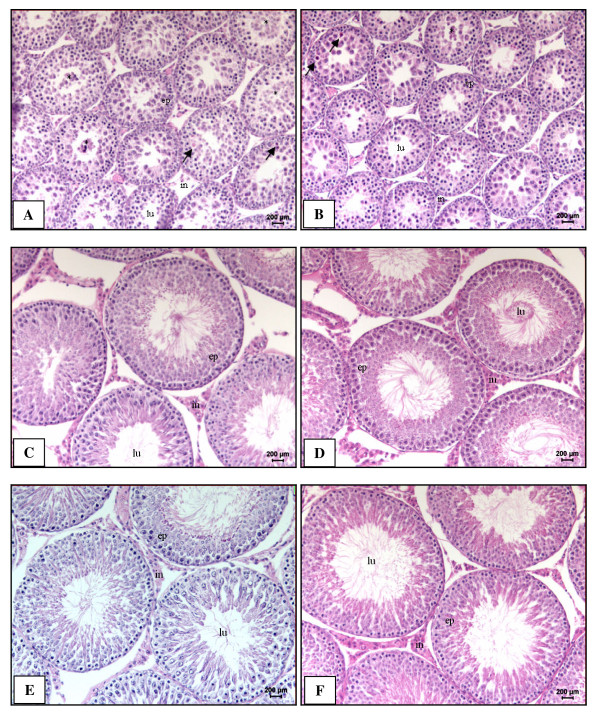
**Histopathological analysis of seminiferous epithelium**. Rats at 30-day-old (A, B), 60-day-old (C, D), 90-day-old (E, F). Photomicrograph of testis sections from HP group (B, D, F) and SC group (A, C, E). H&E stain. 200x magnification. A, B: Note the presence of immature germ cells within the lumen (asterisks) and acidophilic cells (arrows). A-F: tubular lumen (lu), interstitial tissue (in), seminiferous epithelium (ep).

Histological analysis of the testis did not reveal differences between the experimental groups (Figure 1 and Table 3). However, the HC group presented statistically fewer Sertoli cell nuclei in the seminiferous epithelium of 90-day-old rats than the SC group (Table 3). The histological evaluation of the epididymis (data not shown), as well as the stereological analysis of the epididymal cauda (Table 3) did not reveal any alteration that could be attributed to *in utero *protein restriction. The experimental groups did not differ statistically in the degree of maturation of the seminiferous epithelium at any age (data not shown).

**Table 3:** Analysis of testicular histology (30-, 60- and 90-day-old rats), Sertoli-cell-nucleus number (90-day-old rats) and epididymis stereology (60- and 90-day-old rats)

	HC Group			SC Group		
	**30 days** **(n = 10)**	**60 days** **(n = 9)**	**90 days** **(n = 9)**	**30 days** **(n = 10)**	**60 days** **(n = 9)**	**90 days** **(n = 10)**

***Testis***						
Normal tubules (%)	68.0 [63.5-70.7]	98.0 [97.0-99.0]	97.0 [96.0-98.0]	70.0 [66.0-70.0]	97.0 [97.0-99.0]	96.0 [96.0-97.0]
Sertoli cell nucleus Number			10.53 ± 0.13*			11.45 ± 0.13
***Epididymis***						
Epithelium (%)		25.6 [22.6-28.9]	11.9 [8.3-15.0]		24.7 [21.1-27.8]	12.5 [9.8-16.0]
Lumen (%)		46.4 [38.7-50.3]	72.0 [67.9-78.0]		47.6 [41.8 -52.8]	71.4 [66.0-75.9]
Interstice (%)		26.8 [23.8-35.1]	14.9 [11.8-17.9]		29.2 [23.4-33.2]	16.0 [12.2-21.1]

HC group: hypoproteic chow; SC group: standard chow.

Values expressed as mean ± SEM or median and interquartile intervals [Q_1 _- Q_3_]. Mann-Whitney Test. *p < 0.05

Protein restriction *in utero *caused a statistically significant reduction in the number of mature spermatids in the testis, in the daily sperm production (DSP) and in the number of sperm stored in the cauda epididymis. Caput/corpus epididymis sperm number and the epididymal sperm transit time were unchanged (Table 4).

**Table 4:** Sperm parameters (numbers and morphology) of 90-day-old adult male rats

Parameters	HC Group (n = 10)	SC Group (n = 10)
***Sperm counts in the testis***		
Spermatid number (×10^6^)	140.30 ± 5.61*	168.77 ± 8.98
Spermatid number/g organ (×10^6^/g)	116.28 ± 4.31	133.22 ± 8.04
DSP (×10^6^/testis/day)	23.05 ± 0.92*	27.66 ± 1.47
rDSP (×10^6^/day/g)	19.11 ± 0.73	21.83 ± 1.32
***Sperm counts in the epididymis*** *Caput/Corpus*		
Sperm number (×10^6^)	113.95 ± 6.50	123.98 ± 7.34
Sperm number/g organ (×10^6^/g)	386.00 ± 14.35	386.08 ± 17.66
Sperm transit time (days)	4.96 ± 0.32	4.56 ± 0.31
*Cauda*		
Sperm number (×10^6^)	140.09 ± 8.62*	182.12 ± 4.47
Sperm number/g organ (×10^6^/g)	895.93 ± 38.38*	1091.50 ± 44.84
Sperm transit time (days)	6.12 ± 0.35	6.70 ± 0.32
***Sperm morphology***		
^1 ^Normal shaped sperm (%)	94 [92.25 - 94.87]*	96.25 [95.62 - 96.87]
^1^Sperm with cytoplasmatic droplet (%)	70.25 [64.50 - 79.37]*	45.75 [37.13 - 54.87]

HC group: hypoproteic chow; SC group: standard chow.

DSP - Daily sperm production. rDSP - Relative daily sperm production. Values expressed as mean ± SEM. ^1 ^Values expressed as median and interquartile intervals [Q_1 _- Q_3_]. Mann-Whitney Test. *p < 0.05.

**Figure 2:**
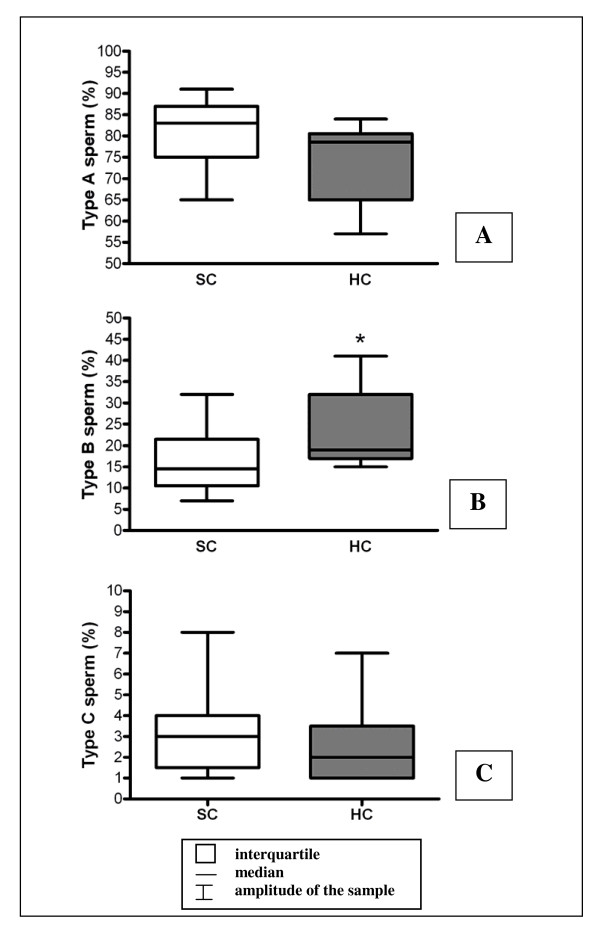
**Sperm motility assessment**. Sperm motility of 90-day-old animals from the standard chow group - SC and hypoproteic chow group - HC (n = 10 per group). Type A sperm (mobile with progressive trajectory), type B sperm (mobile with non-progressive trajectory), type C sperm (immobile). Values expressed as median and interquartile intervals, Mann-Whitney test, *p < 0.05.

Sperm motility evaluation showed a significant increase in the number of type B sperm (mobile, with non-progressive trajectory) in HC when compared to the SC group (Figure 2). The percentage of sperm with abnormal morphology (mainly head abnormalities) was statistically higher in HC than SC (Table 4). In both experimental groups, the cytoplasmic droplet was localized in the middle region of the sperm tail. However, in the HC group the percentage of spermatozoa with cytoplasmic droplet was statistically higher when compared to the SC group (Table 4).

**Figure 3:**
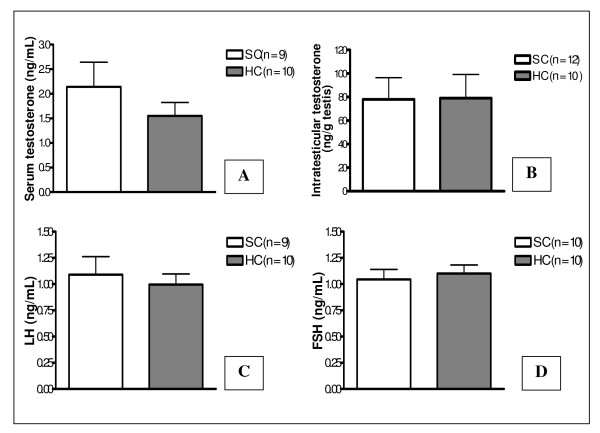
**Serum hormone levels and intratesticular testosterone concentration**. Serum testosterone levels (A), intratesticular testosterone levels (B), serum LH levels (C) and serum FSH levels (D) of 90-day-old animals from the standard chow group - SC and hypoproteic chow group - HC. Values expressed as mean ± SEM. Mann-Whitney test.

Protein restriction *in utero *provoked a non-significant reduction of about 27% in serum testosterone compared to the SC group. Moreover, the concentration of intratesticular testosterone and serum concentrations of FSH and LH were similar between groups (Figure 3).

## Discussion

There is an association between intrauterine growth delay and emergence of diseases in adulthood. This leads to the hypothesis that diseases may have a fetal origin, suggesting that adverse environmental factors such as *in utero *protein malnutrition act in the programming and development of fetal tissue to produce dysfunction and, later, diseases [5,37,38]. Since the end of the 1990s, the possibility of intergenerational effects as a consequence of malnutrition has been considered, with a mistaken initial explanation that these effects were solely due to genetic factors [39]. In 2007, Burdge and co-authors [40] showed that alterations in the methylation status in specific genes of rat pups (F1) exposed to protein restriction during pregnancy can be passed along to subsequent generations, suggesting the importance of epigenetic mechanisms.

In the present study *in utero *protein restriction led to rat body weight reduction, which persisted through different sexual development phases, corroborating previous studies. Melo & Cury [41] found low serum levels of fatty acids and low fat and protein content in the carcasses of pregnant rats that received a hypoproteic diet (6% protein), and concluded that this diet probably affects the supply of circulating nutrients and leads to low pup birth weight. Some authors have shown that protein restriction in pregnant and/or breastfeeding rats can cause metabolic and physiological changes in the offspring, which may be permanent even if the animal has free access to a normal protein diet after weaning [16,42-44].

The absolute and relative weights of organs such as testicles, epididymis, pituitary, seminal vesicle and prostate are useful parameters to evaluate the risks to the male reproductive system. The normal weight of the testis varies little among individuals of the same species, which suggests that its absolute weight is an accurate indicator of gonadal injury, although this parameter may not indicate the nature of the effect [45]. The reduced absolute weight of the prepubertal testicle and adult vas deferens among the HC group can be partly explained by the lower body weight of these animals since, when analyzed in relative terms (g organ/100 g body weight), those same parameters did not differ between experimental groups.

Hormones play a vital role in the establishment and maintenance of male reproductive function [46]. Nutrition is known to cause several effects on the endocrine system [28-31]. Changes in the levels of testosterone, FSH and LH are reported in the offspring of different animal species subjected to malnutrition [16,17,19,26] but, despite a 27% reduction in serum testosterone, our study did not show any statistically significant reduction in sexual hormone levels.

Although the epithelium maturation degree and the results of testicle histological analysis of HC rats in the different developmental phases did not show damage to the seminiferous epithelium, the number of Sertoli cell nuclei was reduced in adult animals. Sertoli cells play a fundamental role in mammalian testicular development and function. In rats, their active proliferation commences during the fetal period and ceases between 2-3 weeks after birth [47].

One of the main functions of the Sertoli cell is to generate a suitable environment for the proliferation and maturation of germ cells. The number of Sertoli cells is a determinant factor for maximum sperm production in adulthood [48]. Thus, changes in the number, structure and/or function of this cell type may damage the germ epithelium and compromise spermatogenesis [49]. Malnutrition from the fetal period to puberty can lead to alterations in the programming of the Sertoli cell number. The low DSP in adults constitutes strong evidence that malnutrition at the beginning of life alters this programming [27]. In our study, the DSP reduction found in HC animals is consistent with the decreased number of Sertoli cells and the 27% reduction in serum testosterone levels. These results corroborate those of Zambrano et al. [16] who subjected rats to *in utero *protein restriction and also observed low sperm count and low fertility rate. These authors concluded that prenatal effects on count and fertility highlight the importance of interactions between different key components in testicular development.

Spermatozoa produced in the testicle are transported to the epididymis, where they undergo a maturation process that confers the motility and capacity needed to fertilize the oocyte [50,51]. Sperm motility is one of the most important parameters used to evaluate the fertile ability of spermatozoa obtained from semen samples both *in vitro *[52] and *in vivo *[53,54]. Changes in motility parameters can lead to sperm inefficiency in penetrating the cervical mucus, thus preventing their access to the oocyte and reducing their fertilizing capacity [45,55-57]. Drop retention in ejaculated sperm may be associated with infertility in several animal species [58]. Zini et al. [59] associated cytoplasmic droplet retention with low sperm motility. Gatti et al. [51] verified that cytoplasmic droplet migration occurs simultaneously with augmentation of sperm motility, but the direct relationship between these two events has not been demonstrated.

The increased number of motile sperm with non-progressive movement and the augmented rate of sperm with cytoplasmic droplet retention in the present study suggest that at least part of the epididymal functions and sperm maturation were affected by protein restriction, although the epididymal structure and the sperm transit time were normal.

Another important parameter for evaluating the fertility potential of males is sperm morphology since sperm alterations may suggest cytotoxic events. In the present study the increased number of spermatozoa presenting head abnormalities may reflect germ cell mutagenicity [60] and deleterious effects of *in utero *protein restriction on the spermatogenic process. Additional studies are necessary to better investigate possible sperm DNA damage and the consequent risk to male fertility potential in present and subsequent generations.

## Conclusion

We can conclude that *in utero *protein restriction, under these experimental conditions, caused growth delay and altered the male reproductive-system programming in rats, leading to a reduction in Sertoli cell and sperm numbers, impairment of sperm motility and morphology, suggesting impairment of sperm quality in adulthood.

## Competing interests

The authors declare that they have no competing interests.

## Authors' contributions

All authors participated in the design and interpretation of the studies, analysis of the data and review of the manuscript; FCT, JEP, FPCP, JAAF conducted the experiments; FCT and WDGK wrote the manuscript. This study represents part of FCT's Masters Thesis presented to the State University of Campinas, under the advisory of WDGK. All authors read and approved the final manuscript.
